# The Superficial Form of Rodent Ulcer

**Published:** 1899-08-26

**Authors:** 


### THE SUPERFICIAL FORM OF RODENT ULCER.

In the last number of the *Archives of Medicine*, Mr. Jonathan Hutchinson points out the importance of recognising as rodent ulcer the very superficial scarring, always, however, with a "rolled edge," which sometimes characterises that disease in its early stage. In the particular case referred to the patient was 53 years old. There was a long elliptical scar on the right lower eyelid. It was not conspicuous, but very definite when looked for. At its margins was a low roll of induration not thicker than a sewing needle, but at most parts quite definite. There was no ulceration whatever, the scar being quite sound, but it was stated that at times a thin crust would form, on the detachment of which a little discharge would be seen. This occurred, however, only at points. The history was one of slow aggression during more than eight years, the border advancing slowly in all directions. In considering such a condition it is necessary to bear in mind its real importance, notwithstanding its apparent insignificance, for the early stage of the disease may be quite as superficial and as slow in progress as in the case described, and yet, finally, ulceration may destroy the greater part of the face.

Mr. Hutchinson drew special attention to the importance of carefully searching for the "rolled edge" in all such cases, for in the very earliest stages of rodent cancer the roll of induration may be very superficial, and may require careful inspection in order to detect it. In some cases there may be doubt as to the diagnosis. "Whenever, however, anything of the nature of a definite rolled edge can be identified which leaves behind it a scar, however slightly marked, there the disease should be recognised, and adequate treatment promptly adopted. It is in this stage that the new growth may be completely eradicated without any severe measures."

By the expression "rolled edge" is meant an elevated, somewhat waved border, quite smooth, and not undermined, and "always somewhat sinuous, in the sense that it is made up of a number of crescents joining at their angles." It is usually free from inflammation and from papillary growth, and although it cannot be asserted that it is absolutely characteristic of rodent cancer "no case of rodent . . . . is without it." In the treatment of these superficial forms Mr. Hutchinson prefers caustics to either scraping or excision, and is accustomed to use fuming nitric acid when there is no appreciable thickening, and Pacquelin's cautery in the more severe cases.

